# Myasthenia gravis in clinical practice

**DOI:** 10.1590/0004-282X-ANP-2022-S105

**Published:** 2022-08-12

**Authors:** Eduardo de Paula Estephan, José Pedro Soares Baima, Antonio Alberto Zambon

**Affiliations:** 1 Universidade de São Paulo, Faculdade de Medicina, Departamento de Neurologia, São Paulo SP, Brazil.; 2 Fundação Faculdade Regional de Medicina de São José do Rio Preto, Hospital de Base, Departamento de Neurologia, São José do Rio Preto SP, Brazil.; 3 Faculdade de Medicina Santa Marcelina, São Paulo SP, Brazil.

**Keywords:** Myasthenia Gravis, Neuromuscular Junction, Neuromuscular Diseases, Neuromuscular Junction Diseases, Practice Guideline, Miastenia Gravis, Junção Neuromuscular, Doenças Neuromusculares, Doenças da Junção Neuromuscular, Guia de Prática Clínica

## Abstract

**Background:**

Myasthenia gravis is largely a treatable disease, but it can result in significant morbidity and even mortality, which can usually be avoided, or at least mitigated, with timely diagnosis and appropriate treatment of the disease. **Objective:** this review aims to summarize the main practical aspects of the diagnostic approach, treatment and care of myasthenic patients.

**Methods:**

The authors performed a non-systematic critical review summarizing the main practical aspects of myasthenia gravis.

**Results:**

Most patients with myasthenia have autoantibodies targeted at acetylcholine receptors or, less commonly, muscle-specific kinase - MuSK. Electrophysiology plays an important role in the diagnosis of neuromuscular junction dysfunction. The central clinical manifestation of myasthenia gravis is fatigable muscle weakness, which can affect eye, bulbar, respiratory, and limb muscles. With rare exceptions, patients have a good response to symptomatic treatment, but corticosteroids and/or immunosuppressants are usually also necessary to obtain good control of the manifestations of the disease.

**Conclusion:**

Knowledge of the peculiar aspects of their clinical and electrophysiological presentations is important for the diagnosis. Likewise, specific treatment and response time to each drug are crucial for proper care.

## RESUMO

**Antecedentes:**

A miastenia gravis é, em grande parte, uma doença tratável, mas pode resultar em significativa morbidade e até mortalidade, que geralmente pode ser evitada, ou pelo menos atenuada, com diagnóstico oportuno e tratamento adequado da doença. **Objetivo:** Esta revisão visa resumir os principais aspectos práticos da abordagem diagnóstica, do tratamento e dos cuidados de pacientes miastênicos.

**Métodos:**

Os autores realizaram uma revisão crítica não sistemática, resumindo os principais aspectos práticos da miastenia gravis.

**Resultados:** A maioria dos pacientes com miastenia apresenta autoanticorpos direcionados aos receptores de acetilcolina ou, menos comumente, quinase músculo-específica - MuSK. A eletrofisiologia desempenha um papel importante no diagnóstico da disfunção da junção neuromuscular. A manifestação clínica central da miastenia gravis é a fraqueza muscular fatigável, que pode afetar os músculos oculares, bulbares, respiratórios e dos membros. Com raras exceções, os pacientes respondem bem ao tratamento sintomático, mas geralmente também são necessários corticosteroides e/ou imunossupressores para se obter um bom controle das manifestações da doença.

**Conclusão:**

O conhecimento dos aspectos peculiares de suas apresentações clínicas e eletrofisiológicas é importante para o diagnóstico. Da mesma forma, o tratamento específico e o tempo de resposta a cada medicamento são cruciais para o cuidado adequado.

## INTRODUCTION

Myasthenia gravis (MG) is largely a treatable disease, but it can result in significant morbidity and even mortality, which can usually be avoided, or at least mitigated, with timely diagnosis and appropriate treatment of the disease. It is a heterogeneous disease from a phenotypic and pathogenetic point of view. The main symptom of myasthenic syndromes in general is fatigable weakness, which can affect virtually any striated muscle. Most of the time, involvement of the extraocular muscles and/or the levator palpebrae are seen at some point in the disease course. Eye symptoms are usually associated with weakness of orofacial, bulbar, limb, neck, and/or respiratory muscles. In this way, the clinical spectrum ranges from a purely ocular form to severe weakness of the limbs, bulbar muscles and respiratory muscles associated to ocular symptoms. Muscle weakness is to due to impairment of neuromuscular junction. Typically, the symptoms are fluctuable, varying in intensity from day to day, or even from hours to hours. The age of onset varies from childhood to late adulthood, with disease peaks in younger adult women and older men^1^ ^,^ ^2^.

Antibodies against acetylcholine receptor (AChR) or other neuromuscular junction targets reduce the number and/or function and/or disorganize their disposition of AChRs at the neuromuscular junction, impairing the safety factor of neuromuscular transmission. In most patients, IgG1 and/or IgG3 attack acetylcholine receptors (AChRs), which leads to fatigable skeletal muscle weakness^2^. The anti-AChRs antibodies production is directly dependent on T cells, with CD4+ T cells stimulating B cells to produce autoantibodies, a process that occurs mainly in an intrathymic environment. Not by chance, most patients with MG have thymic abnormalities, with more than 50% of anti-AChR positive cases having thymic hyperplasia and 10% to 15% thymic tumor, usually thymoma^3^. Carcinoma has also been rarely reported in association with the disease^4^.

Approximately 50% of anti-AChR antibody negative patients have anti-MuSK antibodies^2^ ^,^ ^5^. MuSK is a postsynaptic protein that is critical for the development and maintenance of the neuromuscular junction. Anti-MuSK antibodies are mainly IgG4, not depending on the thymus for their production and not being able to activate complement. In patients with anti-MuSK antibodies, the agrin/MuSK signaling pathway has its functional integrity altered, impairing the normal maintenance of a high density of AChRs in the crests of the neuromuscular junction. Anti-LRP4 antibodies have been reported mainly in Japanese and European patients. The antibodies are of the complement-activating IgG1 type and prevent agrin-induced clustering of AChRs. These antibodies have already been examined by several groups and, in general, their presence in seronegative sera varies widely, and they are associated with pure ocular forms^6^ ^-^ ^9^.

Regardless of the type of antibody, the result of the attack to the neuromuscular junction is a reduced number of functional AChRs with impaired neuromuscular transmission safety factor. Thus, affected endplates become more vulnerable to depletion of acetylcholine stores during repetitive stimulation or sustained muscle contraction. When the amplitudes of some endplate potentials are not sufficient to generate muscle fiber action potentials after repeated/sustained muscle effort, fatigable muscle weakness is seen.

## CLINICAL FEATURES

Clinically, MG is mainly characterized by fatigue and fluctuating and fatigable weakness of the striated muscles. Although any striated muscle can be affected, extraocular, facial, and oropharyngeal muscles are most commonly involved. Fluctuating weakness is characterized by changing in intensity according to the clinical context. Thus, the patient's strength may be better or even normal in some situations and worse or even completely paralyzed in others. Several factors act on the fluctuation of force in myasthenics, such as hormonal variation, ambient temperature, emotional stress, medications that interact with the neuromuscular junction, infections, and physical exercise. When we say that weakness is fatigable, we are referring to the fact that the fluctuation of strength is due to successive contractions of the affected muscle. In fact, exercise and rest are the main influencers of muscle strength in myasthenic patients, being weakness usually prominent after a certain muscle group is used and lessens if that muscle group has some rest. Complaints of altered sensitivity, such as numbness, tingling or pain are not expected, as the symptoms are due to disorders of the junction between the motor nerve and the muscle. That is, sensory nerves are not involved.

Clinically, the myasthenic patient can be classified as having ocular or generalized myasthenia gravis, and generalized myasthenia gravis can be classified as mild, moderate, or severe. Yet, they can be classified as predominantly in bulbar or limb muscles.

Myasthenic weakness typically fluctuates during the day, usually being less severe in the morning and worse as the day progresses, especially after prolonged use of the affected muscles. In two thirds of the cases the symptoms already begin as ocular symptoms, with involvement of the extrinsic eye muscles and/or eyelid levator muscles. In 90% ocular symptoms appear within 2 years of the disease^10^. Weakness remains restricted to the ocular muscles in approximately 10% to 15% of cases (ocular form MG). In the remaining cases, weakness progresses to involve non-ocular muscles during the first 3 years, involving the face, oropharyngeal musculature, and/or limb muscles (generalized MG). Fatigue is also a common symptom of MG that usually manifests from the onset of the disease. However, sometimes the diagnosis can be made difficult by an atypical clinical presentation. Bulbar weakness, with dysarthria/dysphonia, dysphagia, and/or masticatory muscle weakness, may be the main initial symptom in up to 15% of cases, often without prominent ocular symptoms. This type of initial presentation is more common in the elderly. Although more rarely, disease onset is also possible with weakness of an isolated muscle group, such as head extensors, respiratory muscles, phonatory muscles, or even isolated muscle groups of a limb^10^. Large fluctuations in strength can occur due to the presence of myasthenia decompensation factors: emotional stress, systemic illness (especially viral respiratory infections^10^ ^,^ ^11^), hypothyroidism or hyperthyroidism, pregnancy, menstrual cycle, drugs that affect the neuromuscular junction. Regardless of the cause, very important clinical exacerbations of the disease, which lead to respiratory failure or bulbar weakness with severe dysphagia, are called myasthenic crisis, and require specific emergency treatment (immunoglobulin or plasmapheresis)^10^.

Disease course is variable but usually progressive in the first few years, with weakness progressing to involve more muscle during the first 3 years. Maximum weakness sets in during the first year in two-thirds of patients. Even without treatment, the active phase is often followed by an inactive phase, in which fluctuations in strength still occur but are attributable to fatigue, concomitant illness, or other identifiable factors of myasthenia decompensation. 

In anti-MuSK patients, an atypical clinical presentation is more common, in which there is prominent involvement of the bulbar, facial, respiratory and neck muscles, relatively sparing the eye and limb muscles. Clinical pictures similar to those seen in generalized anti-AChR are also common in anti-MuSK cases, and clinical differentiation between these two types of myasthenia is sometimes impossible^12^. However, purely ocular conditions are not expected in an anti-MuSK patient. Symptoms usually start earlier, in the third or fourth decade of life, and women are more commonly affected. Thymic changes are not usually seen. In addition to these characteristics, anti-MuSK positive patients are generally more severe, and myasthenic crises tend to be more frequent in them, sometimes being the first manifestation. These patients tend to respond better to plasma exchange than immunoglobulin for the treatment of myasthenic exacerbations and crises. As for maintenance treatment, they tend to have a good response to rituximab^13^.

In Table 1, the Myasthenia Gravis Foundation of America classification is depicted^14^. 

**Table 1.** MGFA clinical classification^14^.

Class	Clinical features
I	Any ocular muscle weakness; may have weakness of eye closure. All other muscle strength is normal
II IIa IIb	Mild weakness affecting muscles other than ocular muscles; may also have ocular symptoms Predominantly affecting limb, axial muscles, or both Predominantly affecting oropharyngeal, respiratory muscles, or both
III IIIa IIIb	Moderate weakness affecting muscles other than ocular muscles Predominantly affecting limb, axial muscles, or both Predominantly affecting oropharyngeal, respiratory muscles, or both
IV IVa IVb	Severe weakness affecting muscles other than ocular muscles Predominantly affecting limb, axial muscles, or both Predominantly affecting oropharyngeal, respiratory muscles, or both
V	Defined as intubation, with or without mechanical ventilation. The use of a feeding tube without intubation places the patient in class IVb.

## CLINICAL EVALUATION

When examining a patient with myasthenia gravis, or for suspected cases, it is important to demonstrate the fatigability of a given muscle. In this clinical context, it is always useful to test the strength before and after a repetitive effort, particularly in the muscles related to the patient's complaint. For assessment of upper limb strength, the patient may be asked to keep the arms fully extended and raise them above the head for 10 to 15 times, to try to induce fatigable weakness. For weakness of the proximal muscles of the lower limbs, the patient sitting in a chair can be asked to stand up and sit down without the support of the hands for 5 to 10 times.

Some clinical tests and signals were described with the intent to seek evidence of fatigable weakness, and the most used in practice are described below. 

Ice pack test: an ice pack is applied to the closed ptotic upper eyelid for 2 minutes, and then the palpebral fissure is compared with the one present before the test. An improvement in ptosis after ice application (usually 2 mm or more) strongly suggests a myasthenic weakness of the levator palpebrae. In order to facilitate comparison, photo shots with a smartphone, before and after the ice maneuver, can be useful. This test has a sensitivity of more than 80% and a specificity of almost 100%^15^. It is important to avoid leaving the ice pack on for more than two minutes as the test becomes increasingly uncomfortable for the patient and lowering the muscle fiber temperature below 22°C reduces the contractile force of the muscle which can lead to false negatives.

Biefang Test: The patient is asked to squeeze the eyelids tightly for five to ten seconds, which leads to the relaxation of the levator palpebrae superioris muscles, and also actively inhibits it. In addition, the orbicularis oculi muscles, which are responsible for eye closure, can be induced to fatigue. The patient then opens his eyes and fixes his gaze on a target directly in front of him, keeping his gaze fixed. At this moment, a transient decrease in palpebral ptosis can be observed, since the levator palpebrae are more relaxed than the orbicularis oculi (the latter responsible for closing the eyes). If this occurs the test is considered positive^16^.

**Figure 1.**
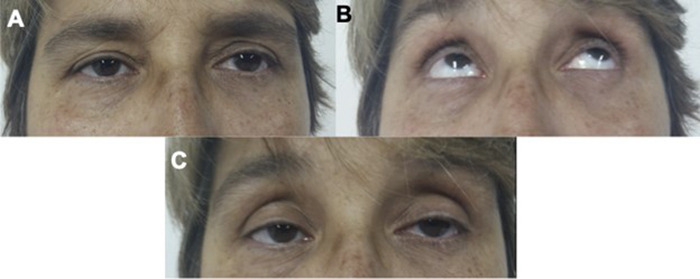
Simpson's Test. A. Patient with asymmetric palpebral ptosis at rest. B. Sustained upward gaze for 2 minutes. C. Increase in ptosis after the maneuver.

Simpson test: It was first described by Alexander Simpson, who observed that in sustained upward gaze, ptosis usually intensifies temporarily^17^. The patient is asked to hold the maximum upward gaze for 1 or 2 minutes, and the behavior of the eyelids is observed. Appearance or intensification of eyelid ptosis characterizes the positive test (Figure 1). Diplopia due to extrinsic ocular muscle fatigue can also be seen.

Cogan's lid twitch sign: it consists of a brief movement of eyelid retraction after the eyes suddenly return to the primary position after a period of downward gaze^18^. The eyelid will contract briefly, becoming more open than in the primary position for a very short period of time, and then settle back into the position it was at rest. In a series of 117 patients, the specificity of this signal was 99% and the sensitivity was 75%^19^.

**Figure 2.**
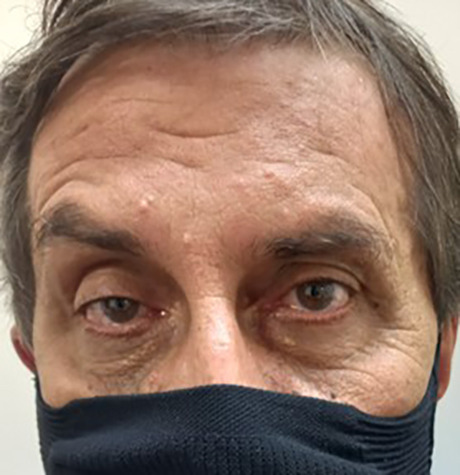
Asymmetrical palpebral ptosis partially compensated by the asymmetric contraction of the frontalis muscle, with uneven elevation of the eyebrows.

Myasthenic characterized by asymmetrical eyelid ptosis, which is partially compensated by the asymmetric contraction of the frontal muscle, raising the ipsilateral eyebrow (Figure 2). During a smile attempt, there is contraction of the medial portion of the upper lip and horizontal contraction of the corners of the mouth without the natural upward curl, producing a "disdain" smile, called a myasthenic smile.

## ELECTROPHYSIOLOGY

Electrophysiology studies (electromyography and nerve conduction study) are performed in patients with suspected neuromuscular junction disorders to confirm defects in neuromuscular transmission. Another aim of the study is to exclude other possible motor unit disorders such as neuropathies, motor neuron disorders, or myopathies. In neuromuscular junction disorders there are no changes in the conventional nerve conduction study, and on electromyography there may be a slight myopathic pattern, especially in cases with more pronounced weakness. There are two classic electrophysiological techniques to evaluate neuromuscular junction function: repetitive nerve stimulation (RNS) and jitter analysis in single-fiber muscle electromyography (SFEMG)^20^. These two cited methods allow diagnosing neuromuscular junction disorders in general and therefore are not specific for MG, although MG is the most common cause of junction disorder^20^.

Common muscles used for repetitive stimulation are Abductor digiti minimi, Tibialis anterior, upper Trapezius, Deltoideus, Orbicularis oculi, and Nasalis. The aim of repetitive nerve stimulation is to observe CMAP (compound muscle action potential) amplitude variation during serial stimuli. Low-frequency (<5-Hz) RNS are indicated to evaluate postsynaptic disorders such as myasthenia gravis. With a low-frequency stimulation, acetylcholine release gradually decreases as presynaptic stores of acetylcholine vesicles are depleted, with a nadir at the fourth or fifth stimulation in a series of stimuli. Positive results are considered when a decrement of 10% or higher is noted in the fourth recorded CMAP. The sensitivity of this study for MG ranges from 53% to 100% for generalized MG, and from 10% to 17% for ocular MG^20^. Proximal muscles seem to present anormal decrement with higher frequency. For maximum diagnostic yield, multiple muscles should be tested, particularly clinically affected muscles^20^. High frequency RNS (30-50 Hz) are best suited to assess presynaptic disorders, such as Lambert-Eaton Myasthenic Syndrome (LEMS). Analogous to the improvement of reflexes after exercise on a clinical basis, a 10-second exercise test can improve CMAP amplitude and is a less harmful stimulus^21^. Positive tests are considered more than 100% increment in CMAP amplitude. Some advocate a 60% increment cutoff, improving sensitivity without losing specificity^22^. It is noteworthy that presynaptic and postsynaptic junction disorders can lead to a significant decrease in low-frequency RNS. However, presynaptic disorders typically have smaller amplitudes before stimulation, which may be an electrophysiological clue to diagnosis.

Neuromuscular junction failure can also be detected on single fiber electromyography, appearing as blockage or increased jitter if the difference in endplate potential amplitude is sufficient to produce delayed depolarization of muscle fibers. During muscle contraction, adjacent fibers of the same motor unit depolarize almost simultaneously. In myasthenia gravis, as a consequence of the variation in transmission time, there may be a delay in depolarization. The range between the depolarization of two fibers of one single motor unit is called jitter. This is the most sensitive method of identifying a neuromuscular junction disorder, and its sensitivity ranges around 98%^23^. A normal jitter test practically excludes an abnormality in the tested muscle. Most common muscles selected are Frontalis, Orbicularis Oculi, and Extensor communis digitorum. Jitter analysis can be done with voluntary contraction of muscle or through a passive analysis. It is important to bear in mind that increased sensitivity of single-fiber EMG comes at the price of reduced specificity. Jitter can be increased in nerve or even muscle disease, when there are secondary motor end plate disorders in these cases notably mitochondrial myopathy and congenital myopathies^20^ ^,^ ^24^.

Pitfalls during the assessment of neuromuscular disorders are low temperature (<33°C) and concomitant use of symptomatic medications prior to the examination. Anticholinesterase inhibitors should be stopped at least 12 hours before the test.

## SEROLOGICAL TESTS

The typical clinical picture, as described above, and the eventual phenomena of neuromuscular junction impairment evidenced in the electroneuromyography (with RNS and/or SFEMG) confirm the diagnosis of MG. The diagnosis can also be made by proving the existence of autoantibodies characteristic of the disease^2^. In addition to diagnostic support, antibody measurement has prognostic value and helps in the therapeutic decision.

In general, an elevated concentration of anti-AChR binding antibodies in a patient with compatible clinical features can also confirm the diagnosis of MG, but normal antibody concentrations do not exclude the diagnosis. Anti-AChR binding antibodies are detected in approximately 80% to 85% of patients with generalized MG, but only in 55% of those with purely ocular symptoms^25^. AChR antibodies are predominantly of the IgG1 and IgG3 subclasses^26^. The predominant mechanism by which antibodies lead to neuromuscular junction dysfunction is activation of the complement cascade. The resulting formation of the membrane attack complex causes damage to the postsynaptic membrane and destruction of synaptic folds that contain AChRs and associated proteins^26^. Other mechanisms of pathogenicity include: antigenic modulation by the binding and crosslinking of AChRs, leading to increased endocytosis and degradation^27^; and blocking of ACh binding to the receptor^28^. Serum concentrations of AChR-binding antibodies vary widely among patients with similar degrees of weakness and cannot reliably predict disease severity. In addition to binding antibodies, there are still two other types of anti-AChR antibodies: blockers and modulators. Blocking antibodies represent a minority of AChR antibodies and usually occur in association with AChR-binding antibodies, and less than 1% of patients without binding antibody have blocking antibodies. Modulator antibodies, on the other hand, occur in about 3% to 4% of patients who are negative for binding antibodies^29^.

A proportion of patients with anti-AChR-negative generalized MG have IgG antibodies against MuSK, a neuromuscular junction protein that plays an important role in the clustering of AChRs. Anti-MuSK antibodies are not normally found in MG positive for anti-AChR antibodies or in ocular MG, although some case reports of patients with ocular MG and anti-MuSK antibodies have been published^30^ ^,^ ^31^. Approximately 50% of anti-AChR negative generalized MG patients have anti-MuSK antibodies^5^, with a female predominance, being 80-85% of MuSK positive patients female^32^. MuSK antibodies mainly belong to the IgG4 subclass, not fixing complement and not strongly activating cell-mediated cytotoxicity^33^. The mechanism by which MuSK antibodies exert their pathogenic effect at the neuromuscular junction is through binding to the Ig-like domain of the protein, preventing its phosphorylation and, subsequently, interrupting the Agrin-Lrp4-MuSK-Dok-7 signaling pathway. 

Anti-striated muscle antibodies were the first autoantibodies discovered in MG. The term anti-striated muscle refers to a class of antibodies against components of skeletal muscle including titin, the ryanodine receptor, myosin, and alpha-actin.They are highly associated with thymoma, being positive in 75% to 80% of MG patients with thymoma, but they are also positive in MG without thymoma, particularly in elderly patients. These antibodies are most useful as a marker for thymoma at the onset of MG before 40^34^. However, they may be a valuable marker for MG in middle-aged or elderly patients with mild disease, in unavailable scenarios of cell-based assays, when it may be the only serological abnormality.

## OTHER EXAMS

Computed tomography should be performed in patients with MG to exclude any presence of thymoma^35^. In these cases, MRI does not improve diagnostic performance. As iodinated contrast agents can exacerbate myasthenic symptoms, the use of these agents is not recommended in the routine investigation of a patient with MG.

Approximately 15% of patients have a second autoimmune disease, which occurs more frequently in patients with early-onset myasthenia gravis and in those with thymic hyperplasia. Thyroiditis is the most common coexisting condition, followed by systemic lupus erythematosus and rheumatoid arthritis. Thus, the baseline thyroid function test should be obtained at the time of diagnosis of MG, and other autoimmune serologies should be considered if clinically indicated^36^.

## DIAGNOSTIC APPROACH

The diagnostic reasoning of myasthenia gravis must always start from a compatible clinical picture. Then, it is recommended that the next step be the AChR-Ab and MuSK-Ab test, with a positive result sufficient for diagnosis^37^. In a seronegative patient, electrodiagnostic tests are the next objective step in diagnosis. The RNS is less sensitive, but more available and highly specific. If the tests are negative, jitter measurement is performed. Electrodiagnostic studies should be directed at clinically involved muscles; it is wise to include as many muscles as possible. If electrodiagnosis confirms postsynaptic disorder, the diagnosis is definite^37^. When the clinical picture is very suggestive, but none of the tests confirm it, the response to pyridostigmine may play a role in supporting the diagnosis. This is especially true in ocular MG or when more accurate antibodies tests are not available. Table 2 shows the main differential diagnoses of myasthenia gravis that should be considered when there is no typical clinical or electrophysiological picture, or when the disease course is refractory to treatment. For seronegative patients with no response with corticosteroids and immunosuppressants, it is important to keep in mind congenital myasthenic syndromes^38^. Receptor deficiency due to *CHRNE* mutations and cases related to *RAPSN* can be clinically very similar to acquired MG^39^ ^,^ ^40^. On the other hand, pure ocular symptoms are not expected to be congenital myasthenic syndromes^41^.

**Table 2.** Differential diagnoses of myasthenia gravis.

Topography of dysfunction	Diseases/Syndromes to consider
Ocular muscles	Graves' ophthalmopathy (with hyperthyroidism); phorias and tropias
Central Nervous System	Brainstem injury (eg, multiple sclerosis, ischemia, mass lesion, Wernicke's encephalopathy), blepharospasm
Peripheral nerve	Microvascular neuropathy, Horner syndrome, Miller Fisher syndrome, Guillain-Barré, focal neuropathies affecting craniobulbar function
Other neuromuscular junction disorders	Botulism, congenital myasthenic syndrome, organophosphate toxicity, Lambert-Eaton syndrome
Myopathies	Chronic progressive external ophthalmoplegia, other mitochondrial myopathies, oculopharyngeal dystrophy, myotonic dystrophy

## TREATMENT

### Symptomatic treatment

MG is treated symptomatically with pyridostigmine, which inhibits acetylcholinesterase at the neuromuscular junction, increasing availability of acetylcholine in the synaptic cleft. In this way, it works as a palliative treatment, which aims to compensate for the lower number of functioning acetylcholine receptors with increased disponibility of acetylcholine in the synaptic cleft. The medicine has few serious side effects and, if effective, works quickly. Starting at 30 mg every 4 hours during the day during waking hours, with the first tablet taken within an hour of waking, is a reasonable strategy for starting use. If necessary, the dose can be increased to a maximum of 480 mg/day, depending on tolerance^42^. Diarrhea, one of the most common side effects, is often self-limiting, but if not, loperamide helps in most cases. A cholinergic crisis, in which weakness is aggravated by increasing doses of pyridostigmine, rarely occurs. 

In cases of ocular myasthenia, treatment is started with symptomatic medication only. If there is no sufficient response, it is recommended to add corticosteroid treatment. In cases of generalized myasthenia, at least in the first years of the disease, it is recommended to start corticosteroids and/or immunosuppressants together with symptomatic patients from the beginning of treatment, except in very mild cases. The dose of pyridostigmine should be adjusted as needed based on symptoms. If a patient is able to discontinue its use or greatly decrease the dose, it may be an indicator that the patient has reached immunosuppressive treatment goals and serves as a useful parameter for the reduction of other therapies.

In the event that it becomes clinically obvious that no response has occurred with pyridostigmine, the medication can be discontinued. Positive anti-MuSK cases tend to have less benefit (or even total lack of benefit) and more side effects with pyridostigmine.

### Corticosteroids and immunosuppressive treatment

Prednisone is very helpful in patients with MG, although patience is required. Average doses of prednisone (about 15-20mg) will help most patients with pure or mild generalized eye conditions in about 3 to 4 months^43^. In more severe cases, higher initial doses are frequently used. If, after 2 to 3 months, symptoms do not improve enough, the dose should be progressively increased until satisfactory control or a dose of 1mg/kg. About 2 to 3 months after satisfactory symptom control, it may be appropriate to start decreasing the dose^44^. Symptoms usually recur at doses lower than 5 mg/day, and most patients need long-term low-dose prednisone^45^. Tapering too quickly or while the patient is still symptomatic will almost always result in a relapse, usually a few months after tapering. On the other hand, if high doses (above 30 mg/day) are used initially, about 40% of patients with MG may initially worsen before they begin to improve, and 10% will worsen significantly. Strategies to avoid this initial worsening of symptoms include starting with low doses (eg, 10 mg/d) with increases every 3 to 5 days in 10 mg steps until reaching the desired dose^44^. The use of IVIg or plasma exchange at the start of prednisone may also prevent initial worsening. Prednisone doses higher than 1 mg/kg/day are rarely necessary, a maximum dose of 0.5 mg/kg/day to 0.75 mg/kg/day is usually used. As for side effects, anticipating the worsening of hypertension and glycemic control is useful and improves patient care. Subjects over 50 years of age who are taking more than 7.5 mg/day for more than 3 months should have osteoporosis prophylaxis at baseline.

Azathioprine can be used in MG, alone as a substitute for prednisone or in conjunction with prednisone in patients who need to reduce the prednisone dose. Azathioprine can still be added to prednisone if satisfactory response with prednisone does not occur within 6 to 9 months or if the patient worsens when already on high dose. The effect of azathioprine generally takes around 3 months to start to be noticed, and it can take 6 to 18 months for the maximum effect to be seen. Therefore, patience is recommended to reduce the prednisone dose after the introduction of azathioprine. The target dose of medication should be between 2.5 and 3 mg/kg per day. A good way to gradually increase the dose is to start with 50mg/day and double the dose every 2 weeks, which would decrease the chance of side effects. Monitoring of liver enzymes (alanine aminotransferase, aspartate aminotransferase and gamma glutamyltransferase) and complete blood count is required monthly. Prolonged use of the medication increases the risk of malignancies, especially of the skin, and patients already at risk of skin cancer must be closely monitored for this, although the absolute risk is low^46^.

In patients who do not respond to or tolerate azathioprine, other immunosuppressive drugs may be used. Evidence from randomized controlled trials supports the use of cyclosporine in MG, but potential serious side effects and drug interactions limit its use^47^. Although evidence from randomized controlled trials has conflicting results with mycophenolate, methotrexate, and tacrolimus in MG, these are widely used, and are recommended in several national guidelines for the treatment of MG^35^ ^,^ ^45^.

 It is estimated that 10-15% of patients fall into the refractory category (no improvement or worsening after the introduction of corticosteroids and at least two other immunosuppressive agents used in adequate doses for an adequate period)^42^. For these patients the following therapies may also be used: immunoglobulin or plasmapheresis chronically; cyclophosphamide; rituximab, eculizumab^45^. Rituximab, for which evidence of efficacy in anti-AChR MG is still being built, should be considered as an early therapeutic option in patients with MuSK-MG who have an unsatisfactory response to initial immunotherapy^45^. This recommendation gained further support in 2017, due to a large blinded multicenter review that compared MuSK-positive MG patients who received rituximab with those who received other immunosuppressive drugs^13^. Besides case series reports, beneficial outcomes were observed in a randomized double blind and placebo-controlled study using high-dose cyclophosphamide infusions^48^. Time-to-improvement ranged from one to three months after treatment onset. Given its toxicity and teratogenic effect, its use can be limited. In 2017, eculizumab was approved by the FDA for MG based on the REGAIN study^49^. Patients included in this double-blind placebo study were anti-AChR myasthenics with a generalized form, defined as refractory. It is currently recommended for refractory cases of MG, and the effect of this medication is perceived earlier than other maintenance therapeutic options, being comparable to immunoglobulin.

### Thymectomy

With rare exceptions, all patients with MG and thymoma must undergo surgery to remove the tumor. Additional thymoma treatment will be dictated by the histological classification and the degree of surgical excision^3^. In non-thymomatous MG (without a diagnosis of thymoma) with the presence of anti-AChR antibodies, thymectomy is performed as an option to minimize the dose or duration of immunotherapy, as an option for cases that have not responded to an initial immunotherapy test, or as an alternative for patients who had intolerable side effects to other therapies^50^. Due to the long delay in onset of effect, thymectomy for MG is an elective procedure. It should be performed when the patient is stable and it is considered safe to undergo a procedure in which postoperative pain and mechanical factors can limit respiratory function, in addition to functional worsening due to metabolic stress^50^. 

Thymectomy may be considered in patients with generalized MG without detectable anti-AChR antibodies. Due to the lack of evidence in these cases, the indication should be more cautious, and reserved for those who do not respond adequately to immunosuppressive therapy. With currently available evidence, thymectomy is not recommended in patients with MuSK, LRP4, or agrin antibodies. For prepubertal patients, the value of thymectomy in the treatment of with MG is unclear, but it may be considered in children with generalized MG with anti-AChR antibody. For children diagnosed with seronegative generalized MG, the possibility of a congenital myasthenic syndrome or other neuromuscular condition should be considered, and evaluation at a specialist neuromuscular disease center is valuable before considering thymectomy.

### Treatment of myasthenic crisis

An impending myasthenic crisis requires hospital admission and close observation of respiratory and bulbar functions. Admission should be in a place with the possibility of transfer to the intensive care unit, in case a patient develops into a manifest crisis. Human immunoglobulin (IVIg) and plasma exchange (PLEX) are used as short-term treatment for these conditions. Corticosteroids or other immunosuppressive agents are often started, or have dosage increased, at the same time to achieve a sustained clinical response. As corticosteroids can cause a transient worsening of myasthenic weakness, it may be appropriate to wait a few days for PLEX or IVIg (2g/kg, divided over 2 to 5 days) to have a beneficial effect before initiating them. The choice between PLEX and IVIg depends on individual patient factors (eg, PLEX cannot be used in patients with sepsis and IVIg cannot be used in renal failure) and the availability of each. Both are likely to be equally effective in treating severe generalized MG, but the effectiveness of IVIg is less certain in mild or ocular MG. Furthermore, PLEX may be more effective than IVIg in anti-MuSK patients. Finally, despite the lack of evidence, there is consensus among experts that PLEX takes effect more quickly^42^.

In conclusion, myasthenia gravis is a highly heterogeneous disease, with different possible pathophysiology and variable severity. Knowledge of the peculiar aspects of their clinical presentations and electrophysiology is important for the diagnosis. Likewise, specific treatment and response time to each drug are crucial for proper care.
